# Ibuprofen and nimesulide derivatives selectively induce apoptosis in HER2-positive breast cancer via inhibition of the PLA₂–COX-2–NF-κB pathway

**DOI:** 10.1007/s11033-026-11835-6

**Published:** 2026-04-24

**Authors:** Egemen Çakırlı, İpek Bedir, Yağmur Biliz, Özgür Yılmaz, Ş. Güniz Küçükgüzel, Dilek Telci

**Affiliations:** 1 Department of Genetics and Bioengineering, Yeditepe University, Istanbul, Türkiye; 2 Department of Chemical Engineering, Faculty of Engineering and Natural Sciences, Istanbul Health and Technology University, Istanbul, Türkiye; 3 TUBITAK Marmara Research Center, Kocaeli Gebze, Türkiye; 4 Department of Pharmaceutical Chemistry, Faculty of Pharmacy, Istanbul University, Istanbul, Türkiye; 5 Department of Pharmaceutical Chemistry, Faculty of Pharmacy, Fenerbahçe University, Istanbul, Türkiye

**Keywords:** HER2-positive breast cancer, Ibuprofen, Nimesulide, Apoptosis, ROS reduction

## Abstract

**Background:**

Chronic inflammation contributes to breast cancer development through the phospholipase A₂ (PLA₂)–cyclooxygenase-2 (COX-2)–nuclear factor κB (NF-κB) cascade, which regulates prostaglandin synthesis, oxidative stress, and transcription of pro-inflammatory and anti-apoptotic genes. This pathway is particularly active in HER2-positive breast cancer, promoting proliferation, invasion, and resistance to apoptosis. Non-steroidal anti-inflammatory drugs such as ibuprofen and nimesulide target COX enzymes and have shown potential in suppressing inflammation-driven tumorigenesis. In this study, we evaluated the anticancer and anti-inflammatory activity of newly synthesized, structurally modified ibuprofen and nimesulide derivatives designed to modulate PLA₂–COX-2–NF-κB axis.

**Methods and Results:**

Cytotoxicity was assessed in HER2-positive breast cancer cells (AU565 and SKBR3) and compared with normal dermal fibroblasts (HDF) and breast epithelial cells (MCF-12A), using WST-1 assays. Apoptosis, cell cycle distribution, caspase-3/7 activation, and ROS generation were analyzed by imaging-based assays, flow cytometry, and fluorescence methods. Gene expression of *PLA2G2A* and *PTGS2* was quantified by qRT-PCR, and NF-κB translocation was analyzed by immunocytochemistry. Two ibuprofen triazole derivative (**D1**) and ibuprofen thioether derivative (**D7**) and one nimesulide derivative (**D8**) significantly reduced cell viability in a dose-dependent manner without affecting normal cells. These derivatives induced G₀/G₁ arrest, caspase-3/7 activation, ROS reduction, and increased late apoptosis. Downregulation of *PLA2G2A* and *PTGS2* expression and inhibition of NF-κB translocation confirmed disruption of the PLA₂–COX-2–NF-κB cascade.

**Conclusion:**

These findings demonstrate that structurally optimized ibuprofen and nimesulide derivatives exert dual anti-inflammatory and anticancer effects in HER2-positive breast cancer by suppressing PLA₂–COX-2–NF-κB pathway and promoting apoptotic cell death.

**Supplementary Information:**

The online version contains supplementary material available at 10.1007/s11033-026-11835-6.

## Introduction

Breast cancer is the most frequently diagnosed cancer among women worldwide, with an estimated 2.3 million new cases and 670,000 deaths in 2022. Global incidence and mortality are projected to rise substantially by 2050, underscoring the need for more effective therapeutic strategies [1, 2]. Breast tumors are classified into basal-like, luminal A, luminal B, HER2-enriched and normal breast-like subtypes based on gene-expression profiles, providing prognostic and therapeutic guidance [3]. HER2-overexpressing breast cancers account for 15–30% of cases and remain clinically challenging due to tumor progression, relapse, metastasis, and therapy resistance [4].

Chronic inflammation is recognized as a driver of breast cancer development and treatment resistance [5]. A central inflammatory network involving phospholipase A₂ (PLA₂), cyclooxygenase-2 (COX-2), NF-κB, and reactive oxygen species (ROS) promotes tumor growth, angiogenesis, immune evasion and metastasis. PLA₂ releases arachidonic acid for COX-2–dependent prostaglandin E₂ (PGE₂) production, which enhances proliferation, survival and immunosuppression [6–8]. PGE₂ further amplifies NF-κB activity, establishing a positive feedback loop that sustains inflammatory signaling [9]. ROS additionally activate NF-κB and increase PLA₂ activity, reinforcing COX-2–mediated prostaglandin synthesis [10, 11]. In HER2-positive tumors, this inflammatory signaling may be particularly pronounced. Constitutive HER2 activation stimulates the RAS/MAPK and PI3K/Akt pathways [12], promoting *PTGS2* (COX-2) transcription and persistent NF-κB activation [13, 14]. These mechanisms link HER2 signaling to activation of the PLA₂–COX-2–NF-κB axis and support targeting of this pathway in HER2-positive breast cancer. Consequently, COX-2 has emerged as a potential therapeutic target, generating interest in non-steroidal anti-inflammatory drugs (NSAIDs) such as ibuprofen and nimesulide. Ibuprofen suppresses prostaglandin synthesis and can inhibit proliferation, induce cell-cycle arrest, activate mitochondrial apoptosis and reduce ROS accumulation by modulating the PLA₂–COX-2–NF-κB–ROS axis [15]. Nimesulide, a selective COX-2 inhibitor, regulates Bcl-2 family proteins, activates caspases and decreases mitochondrial membrane potential, while several derivatives show improved anticancer potency [16, 17]. However, the clinical use of parent NSAIDs is limited by the high doses required to achieve antineoplastic effects. Structural optimization therefore aims to enhance potency and reduce tumor viability at lower concentrations [17, 18]. In this study, novel ibuprofen and nimesulide derivatives previously synthesized and structurally characterized [19–21], were evaluated in AU565 and SKBR3 HER2-positive breast cancer cell lines. Their anticancer and anti-inflammatory activities were assessed through cytotoxicity, apoptosis, ROS and gene-expression analyses to determine their effects on the PLA₂–COX-2–NF-κB–ROS signaling cascade.

## Materials and methods

### Chemistry

All chemicals and solvents used in the syntheses were obtained from commercial sources. The synthesis of the derivatives was performed as described previously [19]. Purification was carried out by silica gel column chromatography (0.063–0.200 mm), and reactions were monitored by thin-layer chromatography (TLC) under 254 nm UV light. Melting points were measured using a Stuart SMP20 Apparatus. ^1^H NMR and ^13^C NMR spectra were recorded at 600 and 150 MHz, in DMSO-d₆ with Me_4_Si as the internal standard. FT-IR spectra were acquired on a PerkinElmer Spectrum BX spectrometer.

### Cell viability and cell cycle assay

AU565 (CRL-2351, ATCC), SKBR3 (HTB-30, ATCC), HDF (CS-201-012, ATCC) and MCF-12A (CRL-3598, ATCC) cells were cultured in RPMI-1640 as described previously [22]. Assay timepoints were selected based on the sequential kinetics of apoptosis: early molecular events (*PLA2G2A*, *PTGS2* expression and ROS generation) were evaluated at 24 h, intermediate signaling events including cell cycle arrest and caspase-3/7 activation between 24 and 48 h, and terminal outcomes such as late apoptosis and maximal cytotoxicity at 72 h.

For viability assays, cells were seeded at 5,000 cells/well and treated with ibuprofen and nimesulide derivatives for 72 h, followed by WST-1 analysis as described previously [22]. For cell-cycle analysis, cells were seeded at a density of 15,000 cells/well in 96-well plates and treated with the IC₅₀ concentrations of each derivative for 24 h. Cells were fixed with 4% paraformaldehyde, permeabilized with Triton X-100, incubated with RNase A, and stained with propidium iodide (PI). Samples were analyzed using the Cytell™ Cell Imaging System (GE Healthcare, UK), with 10,000 cells quantified per sample using the integrated analysis software.

### Annexin V assay

Apoptosis in AU565 and SKBR3 cells was assessed using the Muse^®^ Annexin V & Dead Cell Kit following the manufacturer’s protocol. Cells (3 × 10⁵/well) were seeded in 6-well plates and treated with ibuprofen or nimesulide derivatives or vehicle control (DMSO, 1:1000) for 72 h at toxic concentrations determined by WST-1. Both adherent and floating cells were collected and stained with Annexin V reagent for 20 min at room temperature. Samples were analyzed using a Guava^®^ Muse^®^ Cell Analyzer (10,000 events/sample). Apoptotic nuclear morphology was additionally evaluated by Hoechst 33342 staining (Supplementary Fig. 1).

### Caspase-3/7 activity assay

Caspase-3/7 activity was measured using the Caspase-3/7 Activity Assay Kit (Elabscience, USA). AU565 and SKBR3 cells were seeded at 1 × 10⁶ cells/well in 6-well plates and treated with ibuprofen or nimesulide derivatives or vehicle control for 24 and 48 h (AU565) or 24, 36, and 48 h (SKBR3). Both adherent and detached cells were collected, lysed in ice-cold buffer (70 µL/10⁶ cells), and centrifuged at 11,000×g for 15 min at 4 °C. Protein concentrations were determined by Bradford assay. Lysates were incubated with 2×reaction buffer containing Ac-DEVD-pNA substrate for 2 h at 37 °C, and absorbance was measured at 405 nm using a SpectraMax Paradigm microplate reader.

### Determination of gene expression level and immunocytochemistry

AU565 and SKBR3 cells (3 × 10⁵ cells/well) were treated for 24 h with IC₅₀ concentrations of the derivatives or vehicle and were analyzed for *PLA2G2A* and *PTGS2* (COX-2) mRNA expression by qRT-PCR using a StepOnePlus™ Real-Time PCR System, as described [23]. *PTGS2* primers were forward 5′-GAATGGGGTGATGAGCAGTT-3′ and reverse 5′-CAGAAGGGCAGGATACAGC-3′ with 18 S rRNA as the reference gene. Cells seeded at 2 × 10⁴cells/well in 8-well chamber slides were treated for 24 h with IC₅₀ concentrations of the derivatives or vehicle, and evaluated for NF-κB (p65) nuclear translocation using a p65 antibody (Santa Cruz, USA) as described [23]. Confocal images were acquired using a ZEISS LSM 900 microscope, and nuclear NF-κB fluorescence intensity was quantified per cell using ImageJ/Fiji software.

### ROS assay

Intracellular ROS levels were measured using the DCFDA/H₂DCFDA Cellular ROS Assay Kit (Abcam, UK). Cells were seeded at 25,000 cells/well in 96-well plates and treated for 24 h with IC₅₀ concentrations. After incubation with 20 µM DCFDA for 45 min at 37 °C, cells were washed with PBS, and fluorescence was measured at 485/535 nm as before. Live-cell imaging was performed using a ZEISS Axioscope 5 fluorescence microscope with a FITC filter.

### Statistical analysis

Data are presented as mean ± SD from at least three independent experiments. Parametric group comparisons were performed using one-way or two-way ANOVA with Tukey post hoc tests. Statistical analyses were conducted in GraphPad Prism 10 (GraphPad Software, USA). Differences were considered statistically significant at *P* ≤ 0.05 (*), *P* ≤ 0.01 (**), *P* ≤ 0.001 (***), and *P* ≤ 0.0001 (****).

## Results

### Cytotoxic evaluation of ibuprofen and nimesulide derivatives in HER2-positive breast cancer cells

**Fig. 1:**
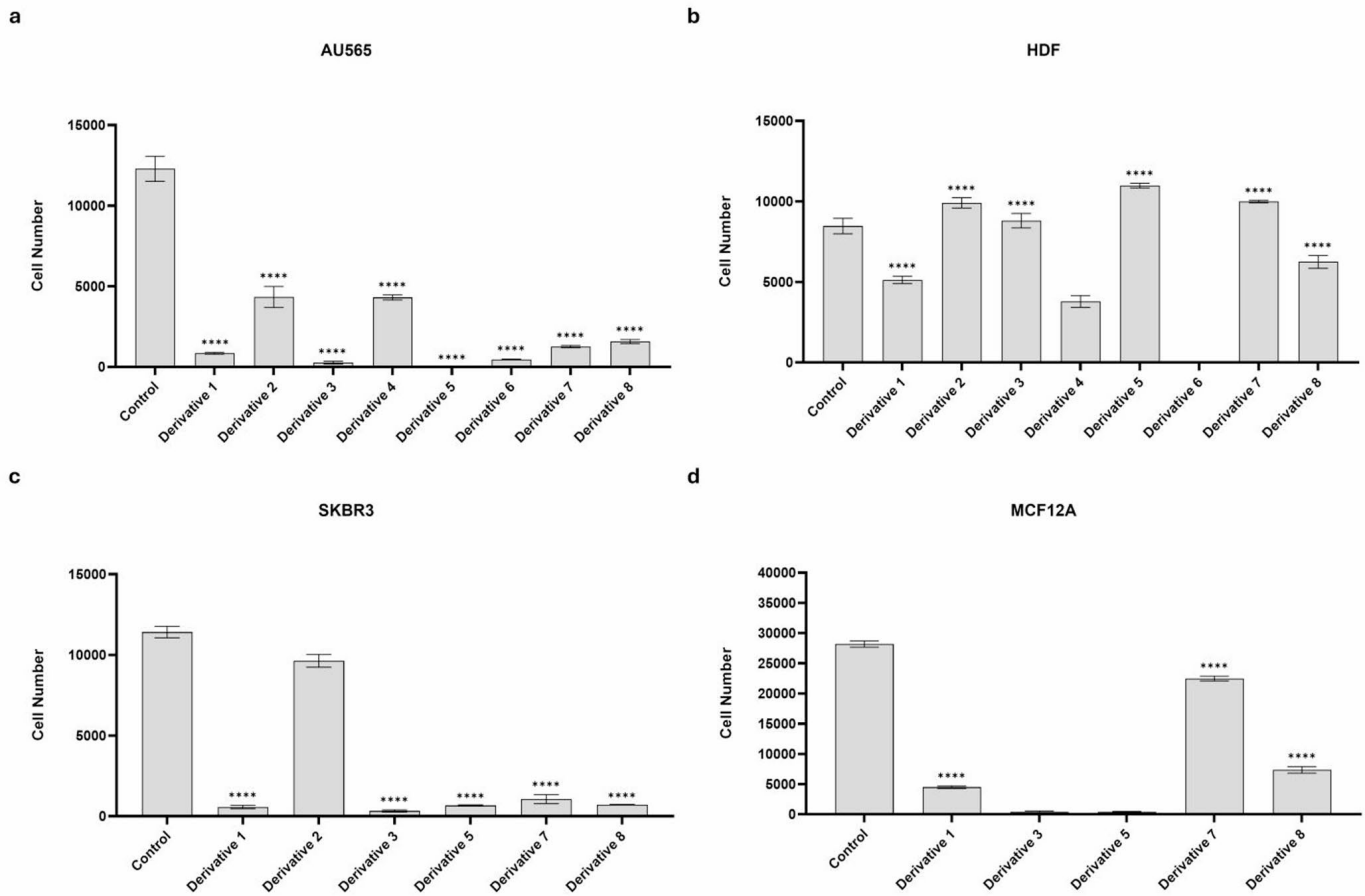
Viability of HER2-positive breast cancer and normal cell lines following treatment with ibuprofen and nimesulide derivatives. Cell viability was assessed by WST-1 assay after 72 h of treatment with selected ibuprofen and nimesulide derivatives in HER2-positive AU565 and SKBR3 breast cancer cells and normal HDF and MCF-12 A cells. (**a**) AU565 breast cancer cells. (**b**) HDF fibroblast cells. (**c**) SKBR3 breast cancer cells. (**d**) MCF-12 A mammary epithelial cells. Data are presented as mean ± SD from three independent experiments (****P ≤ 0.0001 vs. control)

Ibuprofen triazole (**Derivatives; D1-6**), ibuprofen thioether (**D7**) and nimesulide derivative (**D8**) were received as dry powders from TÜBİTAK-MAM and dissolved in DMSO to determine their solubility limits [19–21]. Since DMSO concentrations above 0.1% are toxic to mammalian cells, each derivative was tested at its highest soluble concentration within this limit. The effects on cell viability were first evaluated in AU565 breast cancer cell line using the WST-1 assay (Fig. 1a). Based on the initial screening, derivatives maintaining cell viability above the critical threshold (cell number exceeded the initial seeding density of 5000 cells) were excluded from further analysis. The remaining derivatives were tested on HDF cells to evaluate potential toxicity on normal cells (Fig. 1b). Derivatives that significantly reduced HDF viability were excluded. None of the remaining derivatives except **D4** and **D6** showed cytotoxicity in HDF cells, although **D1** and **D8** induced a cytostatic effect.

The selected derivatives were subsequently evaluated in SKBR3 cells, another HER2-positive breast cancer model, under the same experimental conditions (Fig. 1c). Among the tested derivatives, **D1**, **D3**, **D5**, **D7** and **D8** demonstrated marked cytotoxicity, whereas **D2** exhibited limited activity. Therefore, all selected derivatives except **D2** were further evaluated in the non-tumorigenic mammary epithelial MCF-12A cell line to assess cancer selectivity.

The final selection was based on cytotoxic profiles in MCF-12A cells (Fig. 1d). Through this screening approach, **D1**, **D7** and **D8** were identified as the most selective derivatives (Table 1). The maximum selective concentrations were 70.4 µM for **D1**, 18.9 µM for **D7**, and 86 µM for **D8**. After 72 h-treatment, IC₅₀ values in AU565 cells were, 46.4 µM (**D1)**, 10.5 µM (**D7)** and 8 µM (**D8)**, while in SKBR3 cells, they were 51.6 µM, 13.5 µM, and 26 µM, respectively.

**Table 1:** Comparative cytotoxicity and selectivity profiles of ibuprofen and nimesulide derivatives in breast cancer and normal cell lines

Derivative	Chemical Class	IC_50_ (µM)			Selectivity Index		Cytotoxic/Cytostatic Effect	
		AU565	SKBR3	MCF12A	AU565	SKBR3	AU565	SKBR3
D1	Ibuprofen	46.4	51.6	65.3	1.41	1.27	Moderate Cytotoxic	Moderate Cytotoxic
D7	Ibuprofen	10.5	13.5	45	4.29	3.32	Strong Cytotoxic	Strong Cytotoxic
D8	Nimesulide	8	26	72.7	9.06	2.80	Strong Cytotoxic	Strong Cytotoxic

Selectivity index (SI) values were calculated as the ratio of the IC₅₀ in MCF-12A cells to that in cancer cells [24]. For AU565 cells, the SI values were 1.41 (**D1)**, 4.29 (**D7)**, and 9.06 (**D8)**, while for SKBR3 cells, the respective values were 1.27, 3.32, and 2.80. These findings indicate preferential cytotoxicity of the derivatives toward HER2-positive breast cancer cells.

### Ibuprofen derivatives induced G₀/G₁ phase arrest in both AU565 and SKBR3 cell lines

**Fig. 2:**
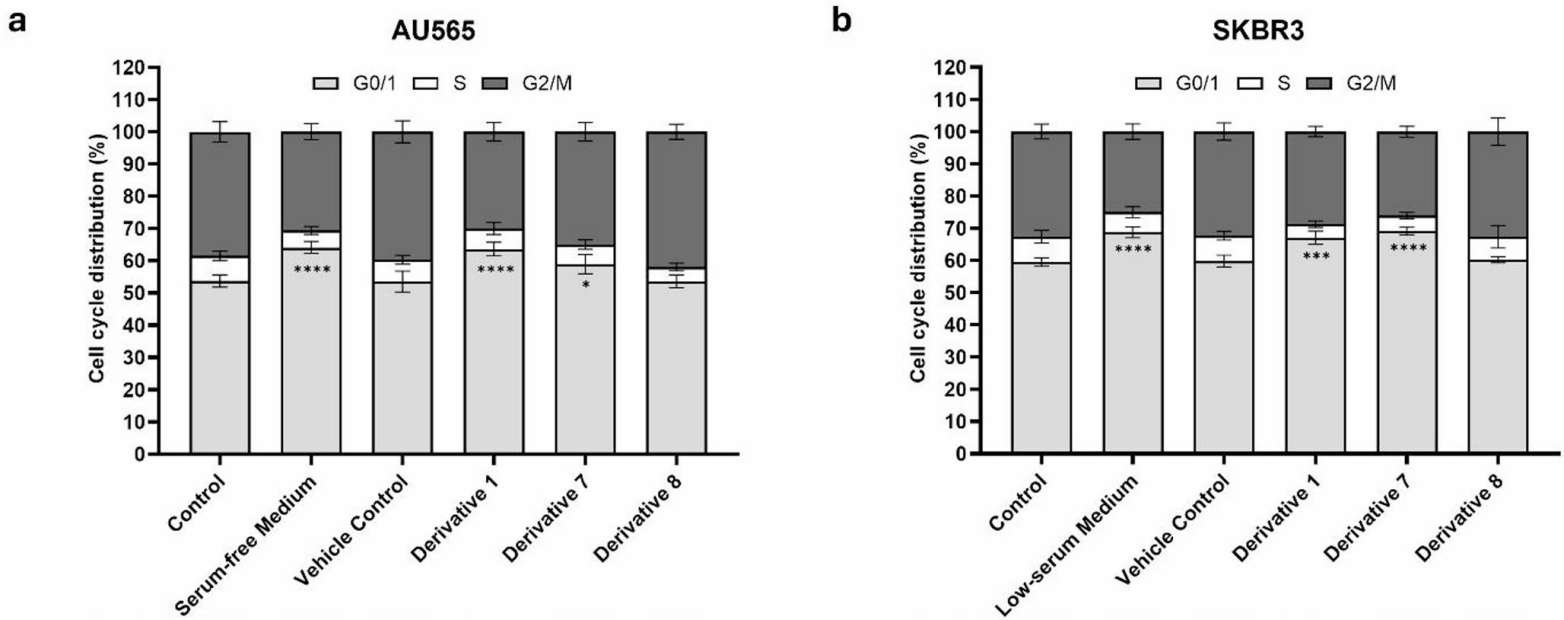
Effects of ibuprofen and nimesulide derivatives on cell cycle distribution in AU565 and SKBR3 cells. (**a**) AU565 and (**b**) SKBR3 cells were treated with **D1**, **D7**, or **D8** for 24 h, and cell cycle phase distributions were determined by Cytell Cell Imaging System (GE Healthcare, UK). Data are presented as mean ± SD from three independent experiments (*P ≤ 0.05, ***P ≤ 0.001, ****P ≤ 0.0001 vs. control)

The effects of ibuprofen and nimesulide derivatives on cell-cycle progression were examined in AU565 and SKBR3 cells after 24-hour treatment. In AU565 control cells, 53.7% of the population was in the G₀/G₁ phase. Treatment with **D1** and **D7** increased this population to 63.6% and 58.9%, respectively. Correspondingly, the S-phase population decreased from 7.8% in control cells to 6.4% and 6.1% following **D1** and **D7** treatment. A reduction in the G₂/M population was also observed, indicating G₀/G₁ phase arrest (Fig. 2a).

In SKBR3 cells, the G₀/G₁ phase increased from 59.5% in controls to 67.1% and 69.2% after treatment with **D1** and **D7**, respectively. The S-phase population decreased from 7.9% to 4.2% and 4.8%, with a concurrent reduction in G₂/M cells (Fig. 2b). Treatment with **D8** produced a modest increase of the G₀/G₁ population and reduction in the S-phase cells, although these changes were not statistically significant (*P* > 0.05) (Fig. 2b).

Together, these results indicate that **D1** and **D7** promote G₀/G₁ arrest and suppress entry into S phase in HER2-positive breast cancer cells.

### Annexin V and caspase-3/7 analyses reveal apoptosis induction in HER2-positive breast cancer cells

**Fig. 3:**
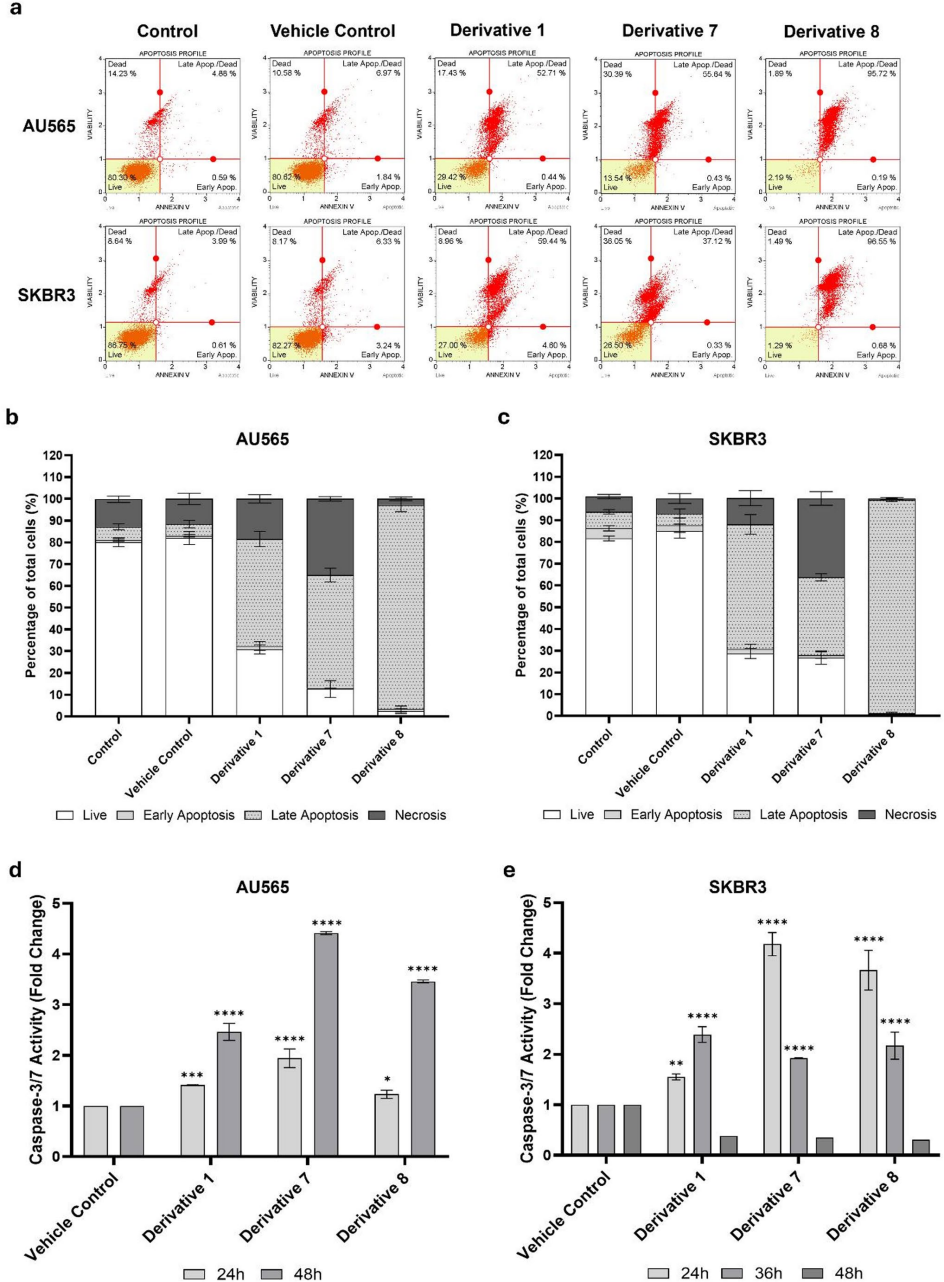
Induction of apoptosis and caspase-3/7 activation by ibuprofen and nimesulide derivatives in HER2-positive breast cancer cells. Apoptosis was evaluated in AU565 and SKBR3 cells following 72 h of treatment with **D1**, **D7**, or **D8**. Annexin V–FITC/7-AAD staining was used to quantify apoptotic cell populations by Guava^®^ Muse^®^ Cell Analyzer (Merck, Germany). (**a**) Representative dot plots showing the apoptotic profiles of control, vehicle control and drug-treated AU565 and SKBR3 cells. Distribution of viable, early-apoptotic, late-apoptotic, and necrotic cell fractions in AU565 (**b**) and SKBR3 (**c**) cells. Caspase-3/7 activity was measured by colorimetric assay in AU565 (**d**) cells at 24 and 48 h, and in SKBR3 (**e**) cells at 24, 36, and 48 h. Results are expressed as fold-change relative to vehicle-treated controls. Data are presented as mean ± SD from three independent experiments (**P ≤ 0.01, ****P ≤ 0.0001 vs. control)

The proapoptotic effects of ibuprofen and nimesulide derivatives were examined in AU565 and SKBR3 cells using Annexin V–FITC/7-AAD staining and caspase-3/7 activity assays (Fig. 3a). Control and vehicle-treated cells showed minimal apoptotic or necrotic populations. In AU565 cells, treatment with **D1** and **D7** induced late apoptosis 49.2 ± 3.5% and 52 ± 3.2% of cells, respectively, with necrosis levels of 18.5 ± 2% and 35 ± 1%. **D8** produced a pronounced apoptotic response, with 93.7 ± 3% late-apoptotic cells and only 2.9 ± 0.9% necrosis (Fig. 3b). In SKBR3 cells, the late-apoptotic fractions reached 57.3 ± 4.5% and 35.8 ± 1.7% after treatment with **D1** and **D7**, with corresponding necrosis levels of 12.1 ± 3.4% and 36.2 ± 3.2%. Treatment with **D8** resulted in a strong apoptotic phenotype, with 98 ± 0.8% late-apoptotic cells and only 0.8 ± 0.4% necrosis (Fig. 3c). Consistent with the Annexin V results, Hoechst 33342 staining showed apoptotic nuclear morphology in AU565 and SKBR3 cells treated with **D1**, **D7**, and **D8**, with chromatin condensation and nuclear fragmentation observed between 24 and 72 h (Supplementary Fig. 1).

Caspase-3/7 measurement further supported apoptosis induction in a time- and derivative-dependent manner. In AU565 cells, caspase-3/7 activity increased 1.4- and 2.5-fold after 24- and 48-hour treatment with **D1**, respectively. Corresponding increases were 1.9- and 4.4-fold for **D7** and 1.2- and 3.5-fold for **D8** compared with vehicle controls (Fig. 3d). Similar trends were observed in SKBR3 cells, where caspase-3/7 activity increased after 24 h-treatment with **D1** (1.6-fold), **D7** (4.2-fold), and **D8** (3.7-fold), followed by moderate activity at 36 h (2.4, 1.9-, and 2.2-fold, respectively). At 48 h, caspase activity declined below control, consistent with late apoptotic regulation and proteolytic turnover of executioner caspases [25] (Fig. 3e).

Together, these findings demonstrate that selected derivatives induce caspase-dependent apoptosis in HER2-positive breast cancer cells.

### Expression of inflammatory mediators *PLA2G2A* and *PTGS2* was downregulated by ibuprofen and nimesulide derivatives

**Fig. 4:**
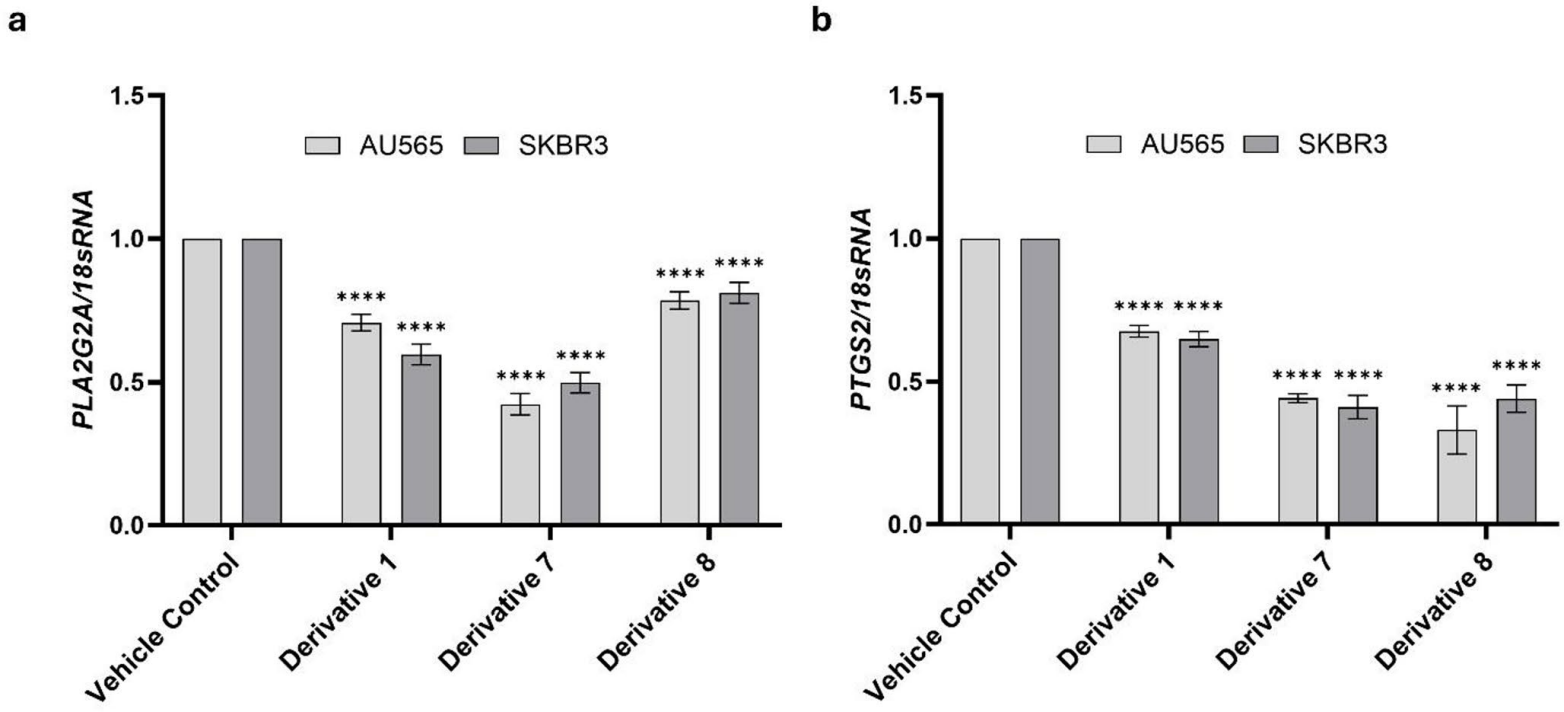
Relative mRNA expression of *PLA2G2A* and *PTGS2* in HER2-positive breast cancer cells following treatment with ibuprofen and nimesulide derivatives. Quantitative real-time PCR (qRT-PCR) analysis was performed on AU565 and SKBR3 cells after 24 h exposure to **D1**, **D7**, and **D8**. (**a**) *PLA2G2A* expression and (**b**) *PTGS2* expression were determined relative to vehicle-treated controls. Gene expression values were normalized to 18sRNA housekeeping gene. Data are presented as mean ± SD from three independent experiments (****P ≤ 0.0001 vs. control)

Given that COX-2-derived eicosanoids such as PGE₂ sustain pro-survival signaling in breast cancer cells [26], we next examined whether the apoptotic effects of **D1**, **D7**, and **D8** were linked to suppression of the arachidonic acid metabolic pathway. To this end, mRNA expression levels of *PLA2G2A* (phospholipase A₂ group IIA) and *PTGS2* (prostaglandin-endoperoxide synthase 2, COX-2) were quantified in AU565 and SKBR3 cells treated with these derivatives using qRT-PCR. In AU565 cells, *PLA2G2A* mRNA levels were significantly downregulated to 0.7 ± 0.03, 0.4 ± 0.04, and 0.8 ± 0.03 following treatment with **D1**, **D7**, and **D8**, respectively. Correspondingly, *PTGS2* expression decreased to 0.7 ± 0.02, 0.4 ± 0.02, and 0.3 ± 0.08 after the same treatments (Fig. 4a). In SKBR3 cells, *PLA2G2A* transcripts were reduced to 0.6 ± 0.04, 0.5 ± 0.04, and 0.8 ± 0.04, while *PTGS2* expression levels were 0.7 ± 0.03, 0.4 ± 0.04, and 0.4 ± 0.05 for **D1**, **D7**, and **D8**, respectively (Fig. 4b). These results validate that ibuprofen and nimesulide derivatives repressed the proinflammatory *PLA2G2A* and *PTGS2* gene expression in AU565 and SKBR3 breast cancer cell lines.

### NF-κB nuclear translocation was suppressed following drug treatment

**Fig. 5:**
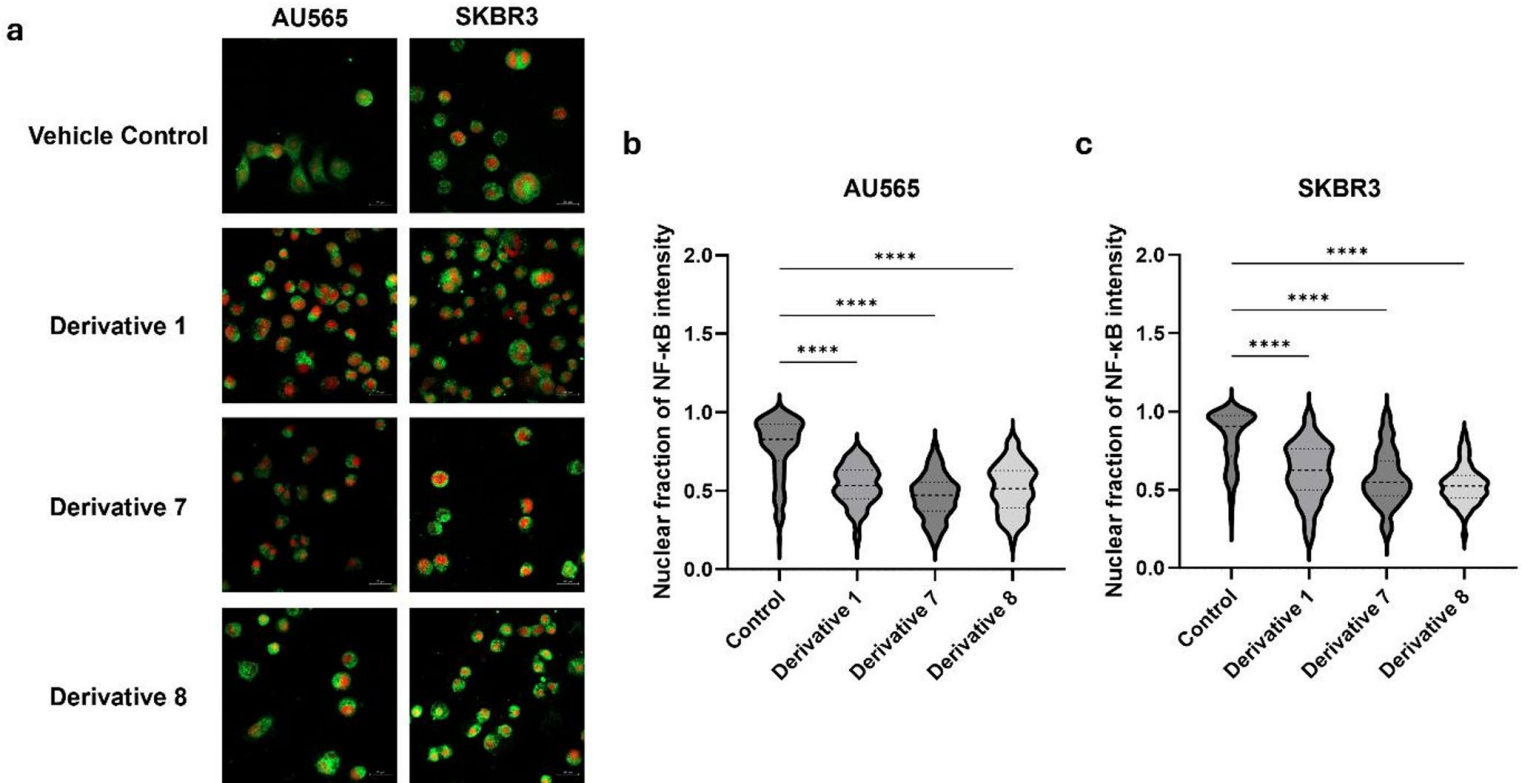
Inhibition of NF-κB nuclear translocation by ibuprofen and nimesulide derivatives in HER2-positive breast cancer cells. Immunocytochemical analysis of NF-κB localization was performed in AU565 and SKBR3 cells after 24 h treatment with **D1**, **D7**, and **D8**. (**a**) Representative confocal fluorescence images showing NF-κB (green, FITC channel) and nuclear counterstaining with PI (red, CY3 channel). Violin plots showing the nuclear fraction of NF-κB fluorescence intensity in AU565 (**b**) and SKBR3 (**c**) cells, quantified on a single-cell basis (*n* ≥ 100 cells per condition, pooled from three independent experiments). Scale bar = 20 μm

Given that NF-κB is a key transcriptional regulator of *PLA2G2A* and *PTGS2*, we next examined whether the observed suppression of this pathway was accompanied by altered NF-κB activity. The subcellular localization of NF-κB was therefore analyzed in HER2-positive breast cancer cells by immunocytochemistry followed by confocal microscopy (Fig. 5a), enabling quantitative assessment of NF-κB nuclear translocation at the single-cell level. The nuclear fraction of NF-κB fluorescence intensity was measured for each cell, and the data were visualized as violin plots to illustrate cell-to-cell variability and treatment-dependent effects. In AU565 cells, quantitative mean fluorescence intensity clearly showed a substantial decline in the nuclear localization of NF-κB. The control group demonstrated a mean fluorescence value of 0.78, while treatments with **D1**, **D7**, and **D8** reduced the nuclear intensity to 0.53, 0.45, and 0.52, respectively; thus, there was less accumulation of NF-κB in the nucleus (Fig. 5b). A comparable trend was observed in SKBR3 cells, where the mean nuclear fluorescence intensity diminished from 0.83 in control samples to 0.62, 0.55, and 0.53 following treatment with **D1**, **D7**, and **D8**, respectively (Fig. 5c). These results show that ibuprofen and nimesulide derivatives effectively prevent NF-κB nuclear translocation in HER2-positive breast cancer cells.

### NSAID derivatives reduced intracellular ROS accumulation

**Fig. 6:**
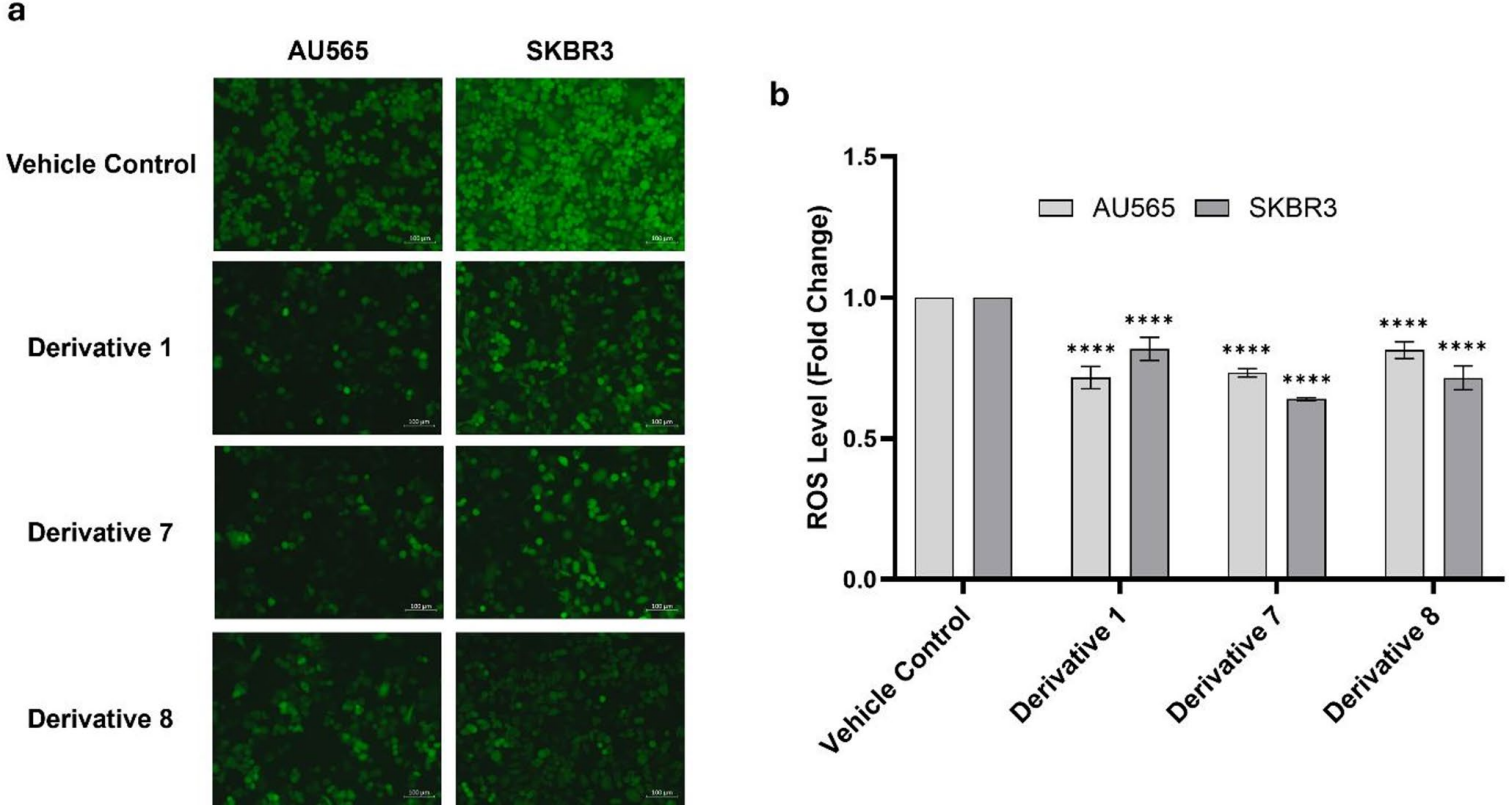
Reduction of intracellular ROS generation by ibuprofen and nimesulide derivatives. (**a**) Representative fluorescence microscopy images for DCFDA-based detection of reactive oxygen species in AU565 and SKBR3 cells treated for 24 h with **D1**, **D7**, and **D8**. (**b**) Quantitative fluorometric evaluation of intracellular ROS expressed as fold change compared to control in AU565 and SKBR3 cells. Scale bar = 100 μm. Data are presented as mean ± SD from three independent experiments (****P ≤ 0.0001 vs. control)

Given that NSAIDs/NSAID-derivatives can modulate redox homeostasis and suppress NF-κB/COX-2 signaling in breast-cancer models [27], we next investigated whether ibuprofen and nimesulide derivatives attenuate intracellular ROS in HER2-positive breast cancer cells. Intracellular ROS levels were evaluated by fluorescence microscopy and fluorometric quantification to determine the modulatory effects of ibuprofen and nimesulide derivatives on oxidative stress in HER2-positive breast cancer cells. Specifically, AU565 and SKBR3 cells treated with **D1**, **D7**, and **D8** using DCFDA-based probe. Fluorescence microscopy images revealed markedly weaker DCF fluorescence in all derivative-treated groups compared with vehicle controls (Fig. 6a). AU565 cells exhibited a 0.7 ± 0.04-, 0.7 ± 0.01-, and 0.8 ± 0.03-fold decrease in ROS levels following treatment with **D1**, **D7**, and **D8**, respectively, compared with control cells (Fig. 6b). Likewise, SKBR3 cells showed a respective 0.8 ± 0.04-, 0.6 ± 0.01-, and 0.7 ± 0.04-fold decrease in ROS levels in response to **D1**, **D7**, and **D8** (Fig. 6b). These findings suggest that ROS production was suppressed effectively by ibuprofen and nimesulide derivatives in HER2-positive breast cancer cells.

## Discussion

Despite progress in surgery, hormone therapy, radiotherapy, and chemotherapy, breast cancer continues to remain a significant health risk for women globally. The ongoing issues of tumor relapse, metastasis, and resistance to treatment highlight the necessity for innovative therapeutic strategies [28].

Chronic inflammation is increasingly recognized as a crucial aspect of breast carcinogenesis. It supports a microenvironment allows such parallel pathways of proliferation, angiogenesis, metastasis, and immune evasion [29]. At the core of this mechanism lies the PLA₂–COX-2–NF-κB axis, which coordinates arachidonic acid metabolism and eicosanoid biosynthesis with the transcriptional regulation of pro-inflammatory cytokines and redox-sensitive pathways govern ROS production [8, 26]. Within this pathway, PLA₂ enzymes catalyze the hydrolysis of membrane phospholipids to release arachidonic acid, which is subsequently converted by COX-2 into prostaglandins such as PGE₂ [6]. PGE₂, in turn, activates downstream signaling cascades involving the transcription factor NF-κB [30]. In this regard, NSAIDs have garnered significant interest due to their ability to inhibit COX enzymes and disrupt prostaglandin-mediated signaling. Consequently, NSAIDs have become a crucial therapeutic strategy in addressing inflammation-related oncogenic pathways.

Ibuprofen is a widely used non-selective COX inhibitor that suppresses prostaglandin synthesis through concurrent inhibition of COX-1 and COX-2, whereas nimesulide preferentially inhibits COX-2 [31]. Both compounds are recognized for their ability to disrupt the PLA₂–COX-2–NF-κB signaling cascade, which is a vital pathway connects inflammation to the survival and proliferation of tumors. Based on this rationale, the derivatives synthesized in this study were designed to retain the anti-inflammatory properties of their parent compounds, while being further optimized to improve their antiproliferative and pro-apoptotic activities in cancer cells.

Several studies have shown that ibuprofen, nimesulide, and their analogues can reduce proliferation by inducing cell cycle arrest and can also trigger apoptosis in breast cancer cell models [27, 32–34]. These findings suggest structural modifications of NSAIDs may enhance their anticancer efficacy by strengthening their ability to arrest the cell cycle and subsequently induce apoptosis, while further suppressing proliferative signaling through modulation of inflammatory and survival pathways. Consistent with this hypothesis, treatment of HER2-positive breast cancer cell lines (AU565 and SKBR3) with the ibuprofen and nimesulide derivatives (**D1**, **D7**, and **D8**) resulted in a marked reduction in cell viability. While ibuprofen typically shows weak cytotoxicity—requiring millimolar concentrations to reach IC₅₀ values (e.g., ~ 5.1 mM in MCF-7 and ~ 1.7 mM in MDA-MB-231 cells) [18] and phospho-ibuprofen derivatives still range between 80 and 200 µM (e.g., ~ 198 µM in AU565 cells) [27], our ibuprofen derivatives displayed substantially lower IC₅₀ values, ranging from 10.5 to 51.6 µM in AU565 and SKBR3 cells. Nimesulide similarly exhibits only moderate potency, with IC₅₀ values of approximately 100–150 µM in breast cancer cells [17], whereas its derivative **D8** shows markedly improved cytotoxicity, with IC₅₀ values ranging from 8 µM to 26 µM for AU565 and SKBR3 cells, respectively. These data suggest that structural refinement confers a substantially enhanced cytotoxic effect.

Cell-cycle analysis demonstrated a prominent accumulation of cells in the G₀/G₁ phase, indicating inhibition of S-phase entry and suppression of proliferative drive. Consistent with our findings, previous studies have shown ibuprofen (1 mM in HTZ-349 glioma cells) and its phospho-derivatives (20 µM in MCF-7 breast cancer cells) induce approximately 60% G₀/G₁ arrest by inhibiting the PI3K/Akt pathway and subsequently downregulating Cyclin D1 [27, 33]. Similarly, nimesulide at 100 µM was reported to cause nearly 62% of G₀/G₁ arrest in AGS gastric cancer cells, while its analogues produced about 50% accumulation of G₀/G₁ in SKBR3 cells at only 5 µM [32, 34]. In our study, treatment of AU565 and SKBR3 cells with ibuprofen derivatives at their IC₅₀ concentrations produced a comparable or even higher degree of G₀/G₁ enrichment at much lower doses. In AU565 cells, **D1** (46.4 µM) and **D7** (10.5 µM) increased the G₀/G₁ fraction to 63.6% and 58.9%, respectively, while in SKBR3 cells **D1** (51.6 µM) and **D7** (13.5 µM) increased it to 67.1% and 69.2%. In contrast, **D8**—which showed lower IC₅₀ values (8 µM in AU565; 26 µM in SKBR3)—produced only modest G₀/G₁ shifts. Taken together, these comparisons suggest, relative to parent ibuprofen and nimesulide, the ibuprofen derivatives **D1** and **D7** achieve robust G₀/G₁ cell-cycle blockade at low-micromolar IC₅₀ values, whereas the nimesulide-based derivative **D8** induces a more modest G₀/G₁ arrest that is comparable to parent nimesulide but achieved at substantially lower IC₅₀ concentrations.

Annexin V–FITC/7-AAD staining revealed increased apoptosis, and caspase-3/7 assays showed time-dependent activation of executioner caspases in AU565 and SKBR3 cells, confirming programmed cell death rather than necrotic cytolysis. Notably, treatment with **D1** (70.4 µM), **D7** (18.9 µM), and **D8** (86 µM) resulted in 70–98% Annexin V–positive cells, demonstrating substantially higher apoptotic induction than their parent compounds. Parent ibuprofen at 750 µM induced only 28% apoptotic and dead cells in MCF-7 cells [35], whereas certain ibuprofen analogues at 160 µM produced nearly 80% Annexin V–positive MCF-7 cells [27]. In contrast, heterosubstituted-dexibuprofen conjugates at 150 µM failed to increase apoptotic fraction or caspase-3/7 activity in MDA-MB-468 cells [36], underscoring the superior pro-apoptotic efficacy of our derivatives. A similar pattern is observed with nimesulide. While nimesulide at 400 µM induced approximately 60% apoptosis in PANC-1 and MCF-7 cells [37, 38], our nimesulide-based derivative **D8** produced equal or greater apoptosis despite acting at an order of magnitude lower concentration. Moreover, 100 µM nimesulide did not elevate caspase-3 activity in pancreatic cancer cells [39], whereas **D8** triggered robust caspase activation in breast cancer models, further highlighting the enhanced potency conferred by chemical optimization.

Given that the parent compounds ibuprofen and nimesulide act on the arachidonic acid cascade at the level of cyclooxygenase enzymes, we next examined whether treatment with the ibuprofen and nimesulide derivatives modulates the expression of key enzymes in this pathway. To this end, *PLA2G2A* and *PTGS2* mRNA levels were quantified in AU565 and SKBR3 cells. At the transcriptional level, all three derivatives significantly downregulated *PLA2G2A* and *PTGS2* in both AU565 and SKBR3 cells. *PLA2G2A* encodes a phospholipase involved in arachidonic acid metabolism and acts as a tumor promoter in breast cancer [6]; its suppression is therefore expected to limit substrate availability for eicosanoid synthesis. Across both AU565 and SKBR3 cells, the derivatives produced an average 20–40% reduction in *PLA2G2A* expression and a 30–60% reduction in *PTGS2* expression. Importantly, these inhibitory effects were achieved within the low-micromolar range for all three derivatives. Compared with our findings, modulation of COX-2 expression by parent ibuprofen and nimesulide generally requires substantially higher concentrations. For instance, an ibuprofen analogue at 80 µM markedly suppressed COX-2 in MCF-7 cells [27], and the COX-2–selective derivative celecoxib produced dose-dependent *PTGS2* downregulation in MCF-7 and MDA-MB-231 cells, reaching maximal suppression around 40 µM [40]. By contrast, several studies report that ibuprofen itself can induce COX-2 at high doses, including a 9-fold increase at 1.5 mM in PC3 cells and a 2–5-fold upregulation of *PTGS2* at 1 mM in head-and-neck and colon cancer cells [41, 42]. Nimesulide has also been shown to reduce expression of COX-2 protein to ~ 50% of control, but only at 100 µM in PANC-1 cells [37]. Collectively, these comparisons showed structural modification of ibuprofen and nimesulide shifts effective *PLA2G2A*/*PTGS2* suppression into the low-micromolar range—markedly lower than concentrations reported for the parent compounds—thereby offering a more efficient means of inhibiting the arachidonic acid–prostaglandin axis in HER2-positive breast cancer cells.

To determine whether the transcriptional suppression of *PLA2G2A* and *PTGS2* translated into functional inhibition of inflammatory signaling, we examined the subcellular localization of NF-κB—a central transcription factor that regulates inflammatory cytokines, cell-cycle genes, and anti-apoptotic pathways in breast cancer and is reinforced through a COX-2–driven feed-forward loop [8, 43]. Immunocytochemical analysis revealed a pronounced reduction in nuclear NF-κB signal in treated AU565 and SKBR3 cells, indicating cytoplasmic retention of the p65/p50 dimer and effective blockade of NF-κB activation. In our study, all three derivatives markedly reduced nuclear NF-κB levels in HER2-positive cells, producing an overall 25–40% decrease and promoting cytoplasmic retention of the p65/p50 complex. This shift effectively kept NF-κB in an inactive state at low-micromolar IC₅₀ concentrations, whereas comparable NF-κB modulation by parent ibuprofen or nimesulide in other tumor models typically required far higher doses. Ibuprofen has been shown to inhibit NF-κB only at high concentrations, reducing p65 levels at 1 mM but not at 130 µM [42, 44], while an ibuprofen analogue required 100 µM to suppress NF-κB activity in MCF-7 cells [27]. Likewise, nimesulide has been shown to inhibit NF-κB nuclear translocation and transcriptional activation, reducing the expression of NF-κB target proteins by approximately 20% at 50 µM in SGC-7901 gastric cancer cells [45].

As oxidative stress is a known upstream activator of NF-κB signaling, intracellular ROS levels were also assessed to determine whether redox modulation contributed to the inhibition. Elevated ROS is typical of proliferating cancer cells and acts as both a damaging oxidant and a signaling intermediate capable of activating IKK and hence NF-κB, and maintaining COX-2 expression and prostanoid biosynthesis [46]. In the present study, all three derivatives markedly reduced intracellular ROS in AU565 and SKBR3 cells, resulting in an overall 20–60% decrease in ROS production. This contrasts with the pro-oxidant behavior reported for the parent NSAIDs: ibuprofen at 750 µM increases ROS in MCF-7 cells, and an ibuprofen analogue at 120 µM induces a four-fold rise in ROS in MCF-7 and SW480 cells [27, 35, 47]. According to experimental models, there have been reports of nimesulide causing injury to mitochondria and hepatotoxicity as a result of inducing oxidative stress and generating superoxides in mitochondria [48, 49]. In contrast to these high-dose pro-oxidant effects, the derivatives in our study reduced ROS at concentrations close to their IC₅₀ values, indicating chemical derivatization shifts ibuprofen and nimesulide toward an antioxidant profile that supports suppression of the NF-κB/COX-2 axis in HER2-positive breast cancer cells. In addition to inhibition of the PLA₂–COX-2–NF-κB axis, NSAIDs have also been reported to modulate stress-activated signaling pathways. In particular, ROS-dependent activation of the p38 MAPK pathway may contribute to cell cycle arrest and apoptosis, suggesting that p38-mediated stress signaling could represent an additional mechanism underlying the observed anticancer effects.

Taken together, our findings highlight the PLA₂–COX-2–NF-κB–ROS axis as a critical molecular vulnerability in HER2-positive breast cancer. The ability of the ibuprofen and nimesulide derivatives to concurrently reduce arachidonic-acid signaling, suppress NF-κB activation, and lower intracellular ROS demonstrates structural optimization can convert classical NSAIDs into multi-target agents with enhanced anticancer potential. By dampening inflammatory and redox-driven survival pathways while promoting apoptosis and cell-cycle arrest, these derivatives effectively shift HER2-positive breast cancer cells toward a low-inflammatory, pro-apoptotic state. This coordinated mechanism supports their further exploration as mechanistically tailored therapeutic candidates for HER2-positive breast cancer.

## Conclusion

The ibuprofen and nimesulide derivatives (**D1**, **D7**, and **D8**) demonstrated potent and selective anticancer activity in HER2-positive breast cancer cells while sparing normal mammary epithelial cells. These derivatives induced robust apoptosis and G₀/G₁ arrest and simultaneously suppressed *PLA2G2A* and *PTGS2* expression, NF-κB nuclear translocation, and intracellular ROS levels. Together, these findings show the derivatives effectively disrupt the interconnected PLA₂–COX-2–NF-κB–ROS axis that sustains inflammatory and survival signaling in HER2-positive breast cancer. By integrating anti-inflammatory and cytotoxic mechanisms, these structurally optimized NSAID-based derivatives provide a mechanistic basis for further therapeutic development. Future studies should evaluate these derivatives in preclinical models to assess pharmacokinetic properties and in vivo antitumor efficacy, as well as explore combination strategies with HER2-targeted therapies.

## Electronic Supplementary Material

Below is the link to the electronic supplementary material.

Supplementary Material 1

## Data Availability

No datasets were generated or analysed during the current study.

## Abbreviations

7-AAD: 7-Aminoactinomycin D
Ac-DEVD-pNA: Acetyl-Asp-Glu-Val-Asp p-nitroanilide
Akt: Protein kinase B
Bcl-2: B-cell lymphoma 2
cDNA: Complementary DNA
COX: Cyclooxygenase
DCFDA: 2′,7′-Dichlorofluorescein diacetate
DMSO: Dimethyl sulfoxide
FITC: Fluorescein isothiocyanate
HER2: Human epidermal growth factor receptor 2
IC50: Half-maximal inhibitory concentration
IgG: Immunoglobulin G
IKK: IκB kinase
NF-κB: Nuclear factor kappa B
NSAID(s): Non-steroidal anti-inflammatory drug(s)
PGE2: Prostaglandin E2
PI3K: Phosphatidylinositol 3-kinase
PLA2G2A: Phospholipase A2 group IIA
PTGS2: Prostaglandin-endoperoxide synthase 2 (cyclooxygenase-2)
qRT-PCR: Quantitative reverse transcription polymerase chain reaction
RNS: Reactive nitrogen species
RNA: Ribonucleic acid
ROS: Reactive oxygen species
WST-1: Water-soluble tetrazolium-1
XIAP: X-linked inhibitor of apoptosis protein

## References

[1] Siegel RL, Giaquinto AN, Jemal A (2024) Cancer statistics, 2024. CA: A Cancer. J Clin 74:12–49. 10.3322/caac.21820 10.3322/caac.21820 38230766

[2] Kim J, Harper A, McCormack V, Sung H, Houssami N, Morgan E, Mutebi M, Garvey G, Soerjomataram I, Fidler-Benaoudia MM (2025) Global patterns and trends in breast cancer incidence and mortality across 185 countries. Nat Med 31:1154–1162. 10.1038/s41591-025-03502-3 39994475 10.1038/s41591-025-03502-3

[3] Turova P, Kushnarev V, Baranov O, Butusova A, Menshikova S, Yong ST, Nadiryan A, Antysheva Z, Khorkova S, Guryleva MV, Bagaev A, Lennerz JK, Chernyshov K, Kotlov N (2025) The Breast Cancer Classifier refines molecular breast cancer classification to delineate the HER2-low subtype. npj Breast Cancer 11:19. 10.1038/s41523-025-00723-0 39979291 10.1038/s41523-025-00723-0 PMC11842814

[4] Semeradt J, Krawczyk O, Krawczyk N, Jędrzejczak P, Kopyciński W, Dębska-Szmich S (2025) Anti-HER2 treatment in solid tumors beyond breast cancer with HER2 overexpression — an exciting new remake of the old target. Oncol Clin Pract 21:279–298. 10.5603/ocp.106480

[5] Xie Y, Liu F, Wu Y, Zhu Y, Jiang Y, Wu Q, Dong Z, Liu K (2025) Inflammation in cancer: therapeutic opportunities from new insights. Mol Cancer 24:51. 10.1186/s12943-025-02243-8 39994787 10.1186/s12943-025-02243-8 PMC11849313

[6] Hidalgo I, Sorolla MA, Sorolla A, Salud A, Parisi E (2024) Secreted phospholipases A2: Drivers of inflammation and cancer. Int J Mol Sci 25:12408. 10.3390/ijms252212408 39596471 10.3390/ijms252212408 PMC11594849

[7] Habanjar O, Bingula R, Decombat C, Diab-Assaf M, Caldefie-Chezet F, Delort L (2023) Crosstalk of inflammatory cytokines within the breast tumor microenvironment. Int J Mol Sci 24:4002. 10.3390/ijms24044002 36835413 10.3390/ijms24044002 PMC9964711

[8] Ma Q, Hao S, Hong W, Tergaonkar V, Sethi G, Tian Y, Duan C (2024) Versatile function of NF-ĸB in inflammation and cancer. Experimental Hematol Oncol 13:68. 10.1186/s40164-024-00529-z 10.1186/s40164-024-00529-z PMC11251119 39014491

[9] Kassab AE (2025) Recent advances in targeting COX-2 for cancer therapy: a review. RSC Med Chem. 10.1039/d5md00196j 40386345 10.1039/d5md00196j PMC12082340

[10] Chaudhary MR, Chaudhary S, Sharma Y, Singh TA, Mishra AK, Sharma S, Mehdi MM (2023) Aging, oxidative stress and degenerative diseases: mechanisms, complications and emerging therapeutic strategies. Biogerontology 24:609–662. 10.1007/s10522-023-10050-1 37516673 10.1007/s10522-023-10050-1

[11] Belahcene S, Kebsa W, Akingbade TV, Umar HI, Omoboyowa DA, Alshihri AA, Mansour AA, Alhasaniah AH, Oraig MA, Bakkour Y, Leghouchi E (2024) Chemical composition antioxidant and anti-inflammatory activities of myrtus communis l. leaf extract: Forecasting ADMET profiling and anti-inflammatory targets using molecular docking tools. Molecules 29. 10.3390/molecules29040849 10.3390/molecules29040849 PMC10893115 38398601

[12] Pan L, Li J, Xu Q, Gao Z, Yang M, Wu X, Li X (2024) HER2/PI3K/AKT pathway in HER2-positive breast cancer: A review. Med (Baltim) 103:e38508. 10.1097/MD.0000000000038508 10.1097/MD.0000000000038508 PMC11175886 38875362

[13] Subbaramaiah K, Norton L, Gerald W, Dannenberg AJ (2002) Cyclooxygenase-2 is overexpressed in HER-2/neu-positive breast cancer. J Biol Chem 277:18649–18657. 10.1074/jbc.M111415200 11901151 10.1074/jbc.M111415200

[14] Cheng X (2024) A comprehensive review of HER2 in cancer biology and therapeutics. Genes 15. 10.3390/genes15070903 10.3390/genes15070903 PMC11275319 39062682

[15] Bülbül B, Ding K, Zhan C-G, Çiftçi G, Yelekçi K, Gürboğa M, Özakpınar ÖB, Aydemir E, Baybağ D, Şahin F, Kulabaş N, Helvacıoğlu S, Charehsaz M, Tatar E, Özbey S, Küçükgüzel İ (2023) Novel 1,2,4-triazoles derived from Ibuprofen: synthesis and in vitro evaluation of their mPGES-1 inhibitory and antiproliferative activity. Mol Divers 27:2185–2215. 10.1007/s11030-022-10551-0 36331786 10.1007/s11030-022-10551-0

[16] Birgül K, Atlıhan İ, Dere D, Yelekçi K, Tiber PM, Orun O, Küçükgüzel ŞG (2025) Investigation of novel nimesulide derivatives against breast cancer. Bioorg Chem 164:108850. 10.1016/j.bioorg.2025.108850 40780124 10.1016/j.bioorg.2025.108850

[17] Su B, Darby MV, Brueggemeier RW (2008) Synthesis and biological evaluation of novel sulfonanilide compounds as antiproliferative agents for breast cancer. J Comb Chem 10:475–483. 10.1021/cc700138n 18380483 10.1021/cc700138n PMC3746990

[18] da Silva EF, Santos PR dos, Fernandes KHA, Freitas D do, de Zanin N, Machado RF, Moura P, de Souza S (2021) Cytotoxic effects of diclofenac and ibuprofen Zinc (II)-nicotinamide ternary complexes in breast cancer cell lines. Braz arch biol technol 64:e21210019. 10.1590/1678-4324-2021210019

[19] Yılmaz Ö, Biliz Y, Ayan S, Çevik Ö, Karahasanoğlu M, Çotuker R, Mert Şahin NM, Gökkaya K, Gülyüz S, Yelekçi K, Küçükgüzel ŞG (2025) Design and synthesis of thiosemicarbazides and 1,2,4-triazoles derived from ibuprofen as potential MetAP (type II) inhibitors. Chemico-Biol Interact 416:111555. 10.1016/j.cbi.2025.111555 10.1016/j.cbi.2025.111555 40345475

[20] Kuloğlu E, Biliz Y, Yılmaz Ö, Koza G, Küçükgüzel ŞG (2024) Synthesis, characterization and antioxidant activities of some novel nimesulide-derived isoureas. In: Alagöz Z, Pabuççuoğlu V (eds) Abstract Book of the 7th National Congress of Pharmaceutical Chemistry, Ege University, Izmir, Abstract P10

[21] Biliz Y, Yılmaz Ö (2024) Synthesis, characterization and antioxidant activitiesof ibuprofen-derived thiosemicarbazides, triazoles and thioethers. In: Alagöz Z, Pabuççuoğlu V (eds) Abstract Book of the 7th National Congressof Pharmaceutical Chemistry, Ege University, Izmir, Abstract S5

[22] Bolat ZB, Nezir AE, Devrim B, Zemheri E, Gulyuz S, Ozkose UU, Yilmaz O, Bozkir A, Telci D, Sahin F (2021) Delivery of doxorubicin loaded P18 conjugated-poly(2-ethyl-oxazoline)-DOPE nanoliposomes for targeted therapy of breast cancer. Toxicol Appl Pharmcol 428:115671. 10.1016/j.taap.2021.115671 10.1016/j.taap.2021.115671 34391753

[23] Bedir I, Ozturk K, Telci D (2025) Impact of PLA2G2A rs11573156 C > G variant on phospholipase expression and metastatic behavior in prostate cancer. Gene 964:149641. 10.1016/j.gene.2025.149641 40550345 10.1016/j.gene.2025.149641

[24] Lica JJ, Wieczór M, Grabe GJ, Heldt M, Jancz M, Misiak M, Gucwa K, Brankiewicz W, Maciejewska N, Stupak A, Bagiński M, Rolka K, Hellmann A, Składanowski A (2021) Effective drug concentration and selectivity depends on fraction of primitive cells. Int J Mol Sci 22:4931. 10.3390/ijms22094931 34066491 10.3390/ijms22094931 PMC8125035

[25] Tawa P, Hell K, Giroux A, Grimm E, Han Y, Nicholson DW, Xanthoudakis S (2004) Catalytic activity of caspase-3 is required for its degradation: stabilization of the active complex by synthetic inhibitors. Cell Death Differ 11:439–447. 10.1038/sj.cdd.4401360 14713960 10.1038/sj.cdd.4401360

[26] Walker OL, Dahn ML, Power Coombs MR, Marcato P (2022) The prostaglandin E2 pathway and breast cancer stem cells: evidence of increased signaling and potential targeting. Front Oncol 11:791696. 10.3389/fonc.2021.791696 35127497 10.3389/fonc.2021.791696 PMC8807694

[27] Sun Y, Rowehl LM, Huang L, Mackenzie GG, Vrankova K, Komninou D, Rigas B (2012) Phospho-ibuprofen (MDC-917) suppresses breast cancer growth: an effect controlled by the thioredoxin system. Breast Cancer Res 14:R20. 10.1186/bcr3105 22293394 10.1186/bcr3105 PMC3496138

[28] Liu S, Zhang X, Wang W, Li X, Sun X, Zhao Y, Wang Q, Li Y, Hu F, Ren H (2024) Metabolic reprogramming and therapeutic resistance in primary and metastatic breast cancer. Mol Cancer 23:261. 10.1186/s12943-024-02165-x 39574178 10.1186/s12943-024-02165-x PMC11580516

[29] Heimes A-S, Shehaj I, Almstedt K, Krajnak S, Schwab R, Stewen K, Lebrecht A, Brenner W, Hasenburg A, Schmidt M (2024) Prognostic impact of acute and chronic inflammatory interleukin signatures in the tumor microenvironment of early breast cancer. Int J Mol Sci 25:11114. 10.3390/ijms252011114 39456897 10.3390/ijms252011114 PMC11507514

[30] Jin K, Qian C, Lin J, Liu B (2023) Cyclooxygenase-2-Prostaglandin E2 pathway: A key player in tumor-associated immune cells. Front Oncol 13:1099811. 10.3389/fonc.2023.1099811 36776289 10.3389/fonc.2023.1099811 PMC9911818

[31] Li J, Shi X, Tang T, Zhou M, Ye F (2024) Research progress on nonsteroidal anti-inflammatory drugs in the treatment of pituitary neuroendocrine tumors. Front Pharmacol 15. 10.3389/fphar.2024.1407387 10.3389/fphar.2024.1407387 PMC11317762 39135798

[32] Chen B, Su B, Chen S (2009) A COX-2 inhibitor nimesulide analog selectively induces apoptosis in Her2 overexpressing breast cancer cells via cytochrome c dependent mechanisms. Biochem Pharmacol 77:1787–1794. 10.1016/j.bcp.2009.03.015 19428334 10.1016/j.bcp.2009.03.015 PMC2954676

[33] Leidgens V, Seliger C, Jachnik B, Welz T, Leukel P, Vollmann-Zwerenz A, Bogdahn U, Kreutz M, Grauer OM, Hau P (2015) Ibuprofen and diclofenac restrict migration and proliferation of human glioma cells by distinct molecular mechanisms. PLoS ONE 10:e0140613. 10.1371/journal.pone.0140613 26485029 10.1371/journal.pone.0140613 PMC4617646

[34] Periasamy J, Muthuswami M, Ramesh V, Muthusamy T, Jain A (2013) Nimesulide and celecoxib inhibits multiple oncogenic pathways in gastric cancer cells. J Cancer Sci Ther/Vol54 05. 10.4172/1948-5956.1000198

[35] Kazberuk A, Chalecka M, Palka J, Surazynski A (2022) Nonsteroidal anti-inflammatory drugs as PPARγ agonists can induce PRODH/POX-dependent apoptosis in breast cancer cells: New alternative pathway in NSAID-induced apoptosis. Int J Mol Sci 23:1510. 10.3390/ijms23031510 35163433 10.3390/ijms23031510 PMC8835909

[36] Thiruchenthooran V, Świtalska M, Maciejewska G, Palko-Łabuz A, Bonilla-Vidal L, Espina M, Luisa Garcia M, Wietrzyk J, Souto EB, Sánchez-López E, Gliszczyńska A (2025) Anticancer activity of phospholipid-dexibuprofen conjugates loaded in nanostructured lipid carriers. Int J Nanomed 20:6999–7019. 10.2147/IJN.S512505 10.2147/IJN.S512505 PMC12134470 40470113

[37] Chu M, Wang T, Sun A, Chen Y (2018) Nimesulide inhibits proliferation and induces apoptosis of pancreatic cancer cells by enhancing expression of PTEN. Exp Ther Med 16:370–376. 10.3892/etm.2018.6191 29896263 10.3892/etm.2018.6191 PMC5995093

[38] Sengel-Turk CT, Hascicek C, Bakar F, Simsek E (2017) Comparative evaluation of nimesulide-loaded nanoparticles for anticancer activity against breast cancer cells. AAPS PharmSciTech 18:393–403. 10.1208/s12249-016-0514-2 27007742 10.1208/s12249-016-0514-2

[39] Eibl G, Reber HA, Wente MN, Hines OJ (2003) The selective cyclooxygenase-2 inhibitor nimesulide induces apoptosis in pancreatic cancer cells independent of COX-2. Pancreas 26:33–41. 10.1097/00006676-200301000-00007 12499915 10.1097/00006676-200301000-00007

[40] Dai Z-J, Ma X-B, Kang H-F, Gao J, Min W-L, Guan H-T, Diao Y, Lu W-F, Wang X-J (2012) Antitumor activity of the selective cyclooxygenase-2 inhibitor, celecoxib, on breast cancer in Vitro and in Vivo. Cancer Cell Int 12:53. 10.1186/1475-2867-12-53 23249419 10.1186/1475-2867-12-53 PMC3558357

[41] John-Aryankalayil M, Palayoor ST, Cerna D, Falduto MT, Magnuson SR, Coleman CN (2009) NS-398, Ibuprofen and COX-2 RNAi produce significantly different gene expression profiles in prostate cancer cells. Mol Cancer Ther 8:261–273. 10.1158/1535-7163.MCT-08-0928 19139136 10.1158/1535-7163.MCT-08-0928 PMC2861287

[42] Grossmannova K, Belvoncikova P, Puzderova B, Simko V, Csaderova L, Pastorek J, Barathova M (2025) Carbonic anhydrase IX downregulation linked to disruption of HIF-1, NFκB and STAT3 pathways as a new mechanism of ibuprofen anti-cancer effect. PLoS ONE 20:e0323635. 10.1371/journal.pone.0323635 40408503 10.1371/journal.pone.0323635 PMC12101644

[43] Kim M-J, Kim H-S, Lee S-H, Yang Y, Lee M-S, Lim J-S (2014) NDRG2 controls COX-2/PGE2-mediated breast cancer cell migration and invasion. Mol Cells 37:759–765. 10.14348/molcells.2014.0232 25256221 10.14348/molcells.2014.0232 PMC4213768

[44] Ouyang N, Ji P, Williams JL (2013) A novel NSAID derivative, phospho-ibuprofen, prevents AOM-induced colon cancer in rats. Int J Oncol 42:643–650. 10.3892/ijo.2012.1756 23291777 10.3892/ijo.2012.1756 PMC3982714

[45] Zhu Z, Liu Y, Cui T, Fei S (2006) The effect of nimesulide on the expression of NF-kB, Bcl-2 and bax in the human gastric cancer SGC-7901 cell line. Chin J Clin Oncol 3:196–201. 10.1007/s11805-006-0118-9

[46] Ma N, Wang Y, Li X, Xu M, Tan D (2025) Reactive oxygen species in cancer: Mechanistic insights and therapeutic innovations. Cell Stress Chaperones 30:100108. 10.1016/j.cstres.2025.100108 40769273 10.1016/j.cstres.2025.100108 PMC12398932

[47] Sun Y, Huang L, Mackenzie GG, Rigas B (2011) Oxidative stress mediates through apoptosis the anticancer effect of Phospho-Nonsteroidal Anti-Inflammatory drugs: Implications for the role of oxidative stress in the action of anticancer agents. J Pharmacol Exp Ther 338:775–783. 10.1124/jpet.111.183533 21646387 10.1124/jpet.111.183533 PMC3164348

[48] Ong MMK, Wang AS, Leow KY, Khoo YM, Boelsterli UA (2006) Nimesulide-induced hepatic mitochondrial injury in heterozygous Sod2 mice. Free Radic Biol Med 40:420–429. 10.1016/j.freeradbiomed.2005.08.038 16443156 10.1016/j.freeradbiomed.2005.08.038

[49] Tay VKS, Wang AS, Leow KY, Ong MMK, Wong KP, Boelsterli UA (2005) Mitochondrial permeability transition as a source of superoxide anion induced by the nitroaromatic drug nimesulide in vitro. Free Radic Biol Med 39:949–959. 10.1016/j.freeradbiomed.2005.05.013 16140214 10.1016/j.freeradbiomed.2005.05.013

