# Supplementary material for: Ibuprofen and nimesulide derivatives selectively induce apoptosis in HER2-positive breast cancer via inhibition of the PLA₂–COX-2–NF-κB pathway
Source: Mol Biol Rep. 2026 Apr 24;53(1):656. doi: 10.1007/s11033-026-11835-6 (PMC13109195; doi:10.1007/s11033-026-11835-6)
Supplement: Supplementary file 1 — Supplementary Material 1 [file 11033_2026_11835_MOESM1_ESM.pdf]

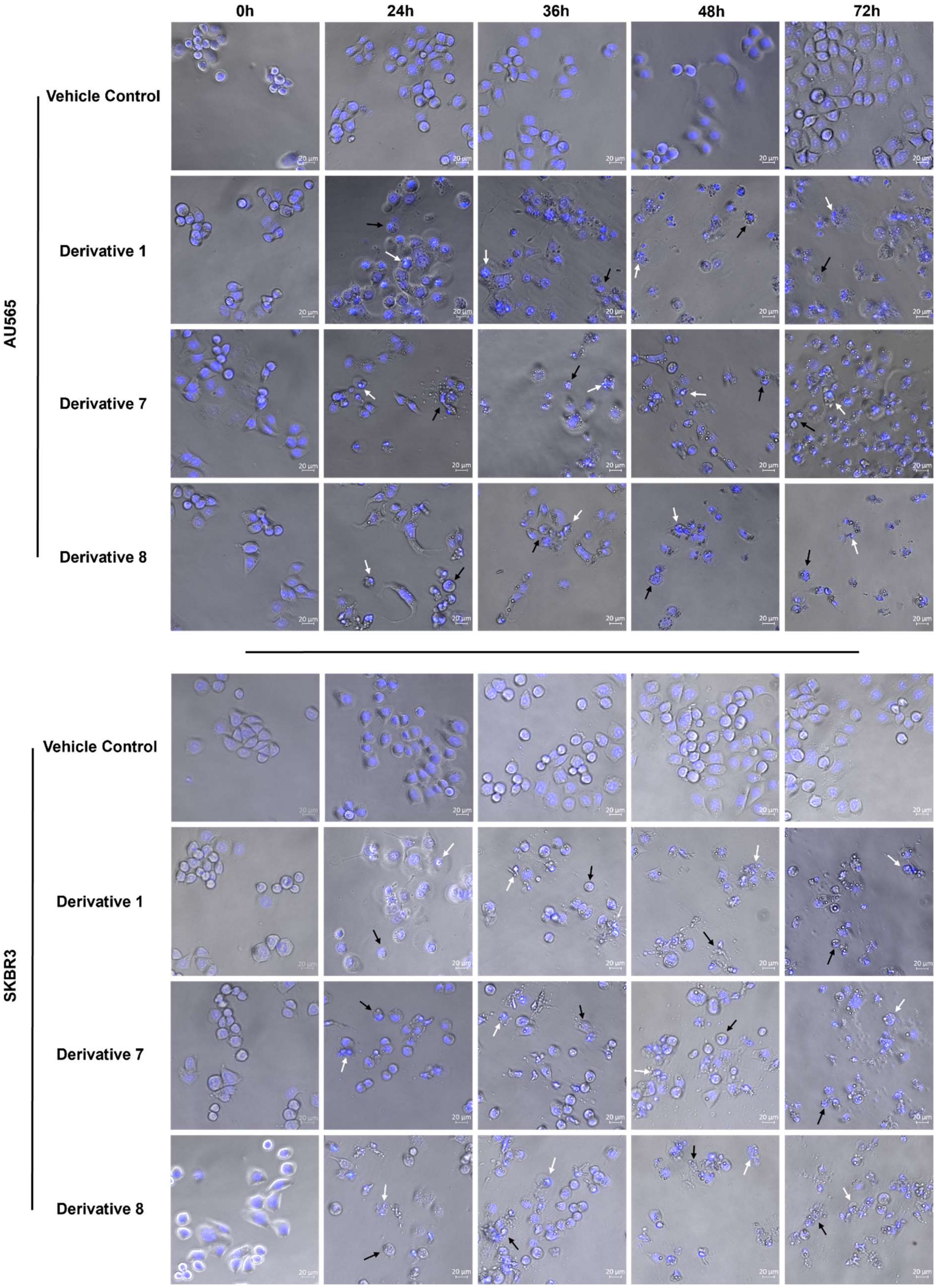
0h
24h
36h
48h
72h
AU565
Vehicle Control
Derivative 1
Derivative 7
Derivative 8
SKBR3
Vehicle Control
Derivative 1
Derivative 7
Derivative 8
20 µm

Supplementary Fig. 1. Qualitative morphological assessment of apoptosis in AU565 and SKBR3 cells by phase-contrast and Hoechst 33342 fluorescence microscopy. Representative merged images were acquired at 0, 24, 36, 48, and 72 hours following treatment with **D1**, **D7**, **D8**, or vehicle control. White arrows indicate condensed or fragmented nuclei, while black arrows indicate apoptotic body-like structures. Scale bar = 20 μm. Cells were seeded in 96-well plates at a density of 5,000 cells/well and treated with ibuprofen or nimesulide derivatives or vehicle control (DMSO, 1:1000). At 0, 24, 36, 48, and 72 h following treatment, Hoechst 33342 was added to a final concentration of 20 μM and cells were incubated at 37 °C for 15 min in the dark, under live-cell conditions. Images were captured using a ZEISS Axioscope 5 fluorescence microscope equipped with a DAPI filter set.
